# Comparative Analysis of Clinical and Radiographic Outcomes Using a Combination of Zinc Oxide and Nano-Hydroxyapatite With Ozonated Oil and Endoflas as an Obturating Material in Primary Teeth: A Prospective Study

**DOI:** 10.7759/cureus.85213

**Published:** 2025-06-01

**Authors:** Souravi Chattopadhyay, Sarmeshta Soni, Deepa Bhatt, Pooja Pani, Aysaiki Maji, Karthikeyan Ramalingam

**Affiliations:** 1 Pediatric and Preventive Dentistry, Awadh Dental College and Hospital, Jamshedpur, IND; 2 Oral Pathology and Microbiology, Malla Reddy Institute of Dental Sciences, Malla Reddy Vishwavidyapeeth, Hyderabad, IND

**Keywords:** antimicrobial, biocompatible, calcium hydroxide, endoflas, nano hydroxyapatite, obturating material, ozonated oil, pulpectomy, resorption, zinc oxide eugenol

## Abstract

Background

A primary tooth plays a vital role in maintaining oral health and general well-being in children. It is important to maintain its position in the dental arch.

Aim

This study aims to compare the clinical and radiographic success of a mixture of zinc oxide and nano-hydroxyapatite with ozonated oil and Endoflas FS (Sanlor, Cali, Colombia) as an obturating material in primary teeth.

Materials and methods

The study sample consisted of 30 human primary teeth treated with pulpectomy in children aged five to eight years, and it was split into two groups. Group A consisted of 15 teeth that were obturated with a mixture of zinc oxide, nano-hydroxyapatite, and ozonated oil (Adc Inclusive Dentozone India, Raigad, India), and group B consisted of 15 teeth that were obturated with Endoflas FS endodontic sealer. Both the groups were observed based on clinical criteria including presence/absence of pain, swelling, mobility, and tenderness on percussion and radiographic criteria including resorption of the material along with physiological resorption of the root, deviation of the path of eruption of the underlying permanent teeth, and resorption of over-pushed material (if any) at the end of the three-month, six-month, and nine-month periods. Statistical analysis was done using the chi-square test using SPSS version 24 (IBM SPSS Statistics for Windows, IBM Corp., Armonk, NY) to see the significant difference (p < 0.05) of each group.

Result

At the end of nine months, both group A and group B showed a 100% success rate clinically, whereas radiographically, group B showed a 100% success rate and group A showed an 80% success rate, with a significant difference of p < 0.05.

Conclusion

Based on the current study, after the clinical and radiographic evaluation for nine months, Endoflas showed betterresults than the mixture of zinc oxide, nano hydroxyapatite, and ozonated oil.

## Introduction

Pediatric oral health is a crucial aspect of dentistry. Nowadays, parents are more careful about their children’s dental health. Dental caries is one of the most common reasons to visit a dentist [1]. A primary tooth plays a vital role and has many functions. It acts as a space maintainer, helping with mastication, speech, and esthetics [2]. Dental caries can be initially treated with restoration, but untreated dental caries may result in pulpal complications. Though not all pulpally involved teeth can be preserved, under certain conditions, such teeth can be treated using pulpectomy [2,3].

Proper biomechanical canal preparation followed by obturation with endodontic filling material is important for the success of pulpectomy. Obturating material plays a very vital role in the success of pulpectomy [3]. Various obturating materials are studied and researched for the obturation of deciduous teeth. Since 1930, zinc oxide eugenol has been the ideal material for obturating root canals in primary teeth due to its antibacterial properties, anti-inflammatory properties, ease of availability, and cost-effectiveness [3,4]. It has disadvantages: it causes a periapical reaction, has limited antibacterial properties, and fails to resorb if extruded from the periapical region [4,5]. Calcium hydroxide plays different roles in endodontics; one of them is a direct pulp capping agent. It was introduced by Herman in 1920. Though calcium hydroxide has advantageous properties, one of the major disadvantages is that it gets resorbed inside the canal rapidly before physiologic resorption of the root, leading to the hollow tube effect [5]. The most commonly used obturating material is zinc oxide eugenol, and its combination with calcium hydroxide and iodoform paste [5,6].

In recent practice, Endoflas (Sanlor, Cali, Colombia) is considered to be the best obturating material, which is biocompatible and resorbs along with the physiologic resorption of the root [4,5]. Endoflas has its drawbacks, too; the eugenol irritates the periapical area if extruded out, and the teeth become discolored. Hence, there is a need to provide better and ideal obturating material for primary teeth [6]. The other obturating materials include iodoform-based pastes, a combination of iodoform and calcium hydroxide, Maisto paste, Guedes-Pinto paste, Calen paste, etc. [5,6]. For decades, hydroxyapatite has been used in orthopedics and dentistry and is well-researched. Hydroxyapatite, due to its osteoconduction and osseointegration properties, is used as a bone graft to reconstruct bone defects [7]. In clinical dentistry, nanohydroxyapatite is used in dentine sensitivity, caries prevention, and bone reconstruction [7,8]. It is a biomimetic agent, and in combination with zinc oxide, it shows antibacterial properties against *Escherichia coli* and *Staphylococcus*. Due to the above beneficial properties of hydroxyapatite, it is used as one of the constituents of obturating material in primary teeth [8].

Research on different oils has been revived and is recognized for its antimicrobial properties. Ozonated oil is composed of ozone and vegetable oil. It has a role in dental caries, endodontics, periodontics, hypersensitivity, etc. Ozonated oil has strong antibacterial properties, is biocompatible, nontoxic, and has high viscosity [9]. Considering the advantageous properties of nanohydroxyapatite, zinc oxide, and ozonated oil, a study was designed using the mixture of zinc oxide, nano-hydroxyapatite (nHAp), and ozonated oil as an obturating material for primary teeth.

This study was done to observe and compare the clinical and radiographic success of a combination of zinc oxide,nHAp, and ozonated oil with Endoflas FS used for obturation of primary teeth.

## Materials and methods

This hospital-based prospective study from the pediatric dental department compared two obturating materials on primary teeth for three months, six months, and nine months. The sample age group was selected based on the availability of patients, their cooperation during observation, and who could be communicated with for further observation. The sample comprised 30 human deciduous teeth in children aged five to eight years visiting the dental outpatient department for dental treatment at Awadh Dental College and Hospital. The samples were split into two groups: group A, where the obturating material was a mixture of zinc oxide powder, nHAp with ozonated oil, and group B, where the obturating material was Endoflas. The ethical clearance was obtained from the ethical committee of Awadh Dental College and Hospital with approval number ECR/1621/INST/JH/2021 and CDSCO, New Delhi, with registration no. MC/1843/22.

The inclusion criteria for the study are pulpectomies done by pediatric dentists on teeth with irreversible pulpitis. The postgraduate students of the pediatric dentistry department did the procedures. The exclusion criteria were external cases already treated outside the hospital, pulpectomies performed by undergraduates, pulp therapy other than pulpectomy, children with a medical history, and those with special needs. 

The pulpectomies were performed in a single visit by a single operator. In all the samples, the biomechanical preparation was by the step-back method. The obturation was done with the manual Lentulo spiral in all the samples, followed by permanent restoration. The teeth were clinically and radiographically evaluated postoperatively for three months, six months, and nine months based on the following criteria: clinical criteria, presence/absence of pain, swelling, mobility, and tenderness on percussion.

The radiographic criteria (RVG) are resorption of the material along with physiological resorption of the root, resorption of material before the resorption of root, resorption of root before resorption of material, deviation of the path of eruption of the underlying permanent teeth, and resorption of over-pushed material (if any). Overfilling of the root canal was also performed on four teeth among both groups to observe any variations between the two materials.

All the samples in groups A and B were observed at three months, six months, and nine months postoperatively. The results were tabulated on a Microsoft Excel sheet (Microsoft® Corp., Redmond, WA); statistical analysis was done with the help of SPSS version 24 (IBM SPSS Statistics for Windows, IBM Corp., Armonk, NY). A chi-square test was performed to see the significance of each group, with a p-value of <0.05.

## Results

The study sample comprised a total of 30 human primary teeth already treated with pulpectomy in patients in the age group of five to eight years visiting the dental outpatient department at the hospital. After completion of pulpectomy, clinical signs and symptoms were tabulated. It was found that both groups A and B showed negative signs of swelling, pain, or tenderness on percussion at the end of three, six, and nine months of observation. Both of the groups showed a 100% clinical success rate. No significant difference was found.

**Figure 1:**
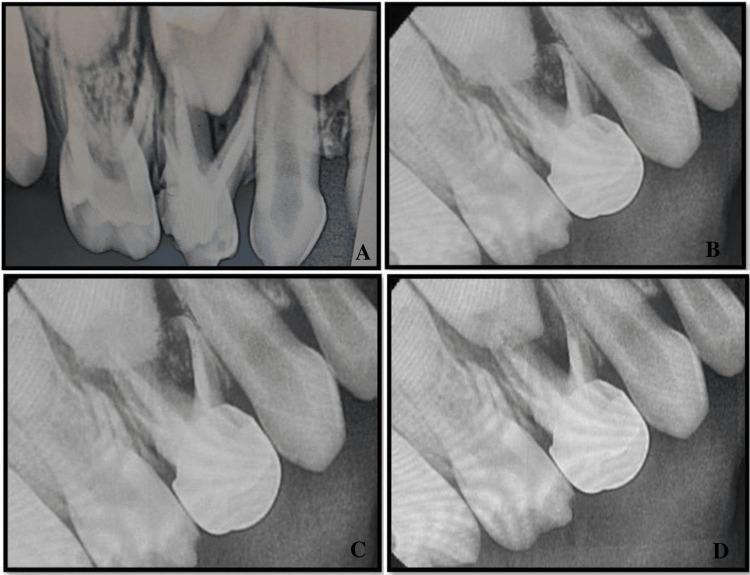
Radiographic assessment (A) Immediate postoperative radiograph. (B) Postoperative radiograph at three months. (C) Postoperative radiograph at six months. (D) Postoperative radiograph at nine months.

Radiographic evaluation of obturated teeth was done among both groups (Figure 1), and the findings were tabulated.

Table 1 presents the distribution of the sample size between two groups, A (n = 15) and B (n = 15), and the distribution of resorption of filling materials in different groups in different follow-ups. The difference among the groups was statistically significant (p < 0.05). The degree of freedom (df) was 1, and the effect size was 0.037. In group A (zinc oxide + nHap + ozonated oil), at the end of three months, all the teeth showed resorption of material and root at the same time. At the end of six months, only in two cases (13.3%) was the material resorbed faster than the tooth, leaving a void in the canal, and the same was seen in three cases at the end of nine months out of 15 samples. So out of 15, only 12 teeth showed resorption of filling material at the same rate as the resorption of the root.

**Table 1:** Distribution of resorption of filling material in the groups in different follow-ups

Follow-up	Group A (n = 15)			Group B (n = 15)			p-value
	Resorption of root equal to filling material	Resorption of rootless than filling material	Resorption of root greater than filling material	Resorption of root equal to filling material	Resorption of rootless than filling material	Resorption of root greater than filling material	
Three months	15 (100%)	0 (0.0%)	0 (0.0%)	15 (100%)	0 (0.0%)	0 (0.0%)	<0.05
Six months	13 (86.7%)	2 (13.3%)	0 (0.0%)	15 (100%)	0 (0.0%)	0 (0.0%)	
Nine months	12 (80.0%)	3 (20.0%)	0 (0.0%)	15 (100%)	0 (0.0%)	0 (0.0%)	

**Figure 2:**
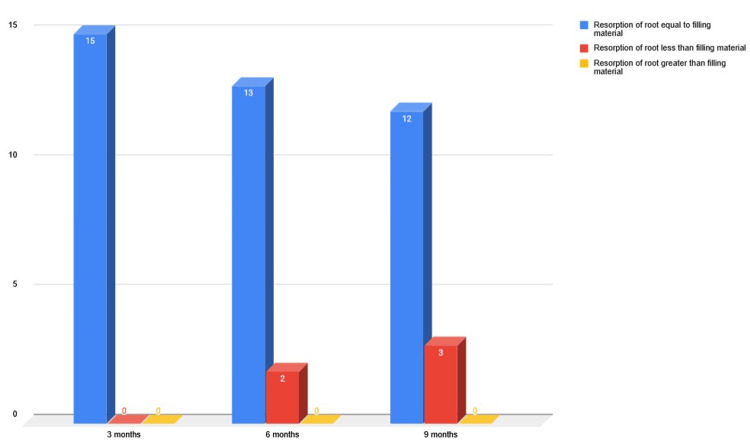
Graph showing the difference in resorption of filling material

In group A (zinc oxide + nHAp + ozonated oil), at the end of six months, only in two cases, the material resorbed fasterthan the tooth, leaving a void in the canal, and the same was seen in three cases at the end of nine months out of 15samples (Figure 2).

**Figure 3:**
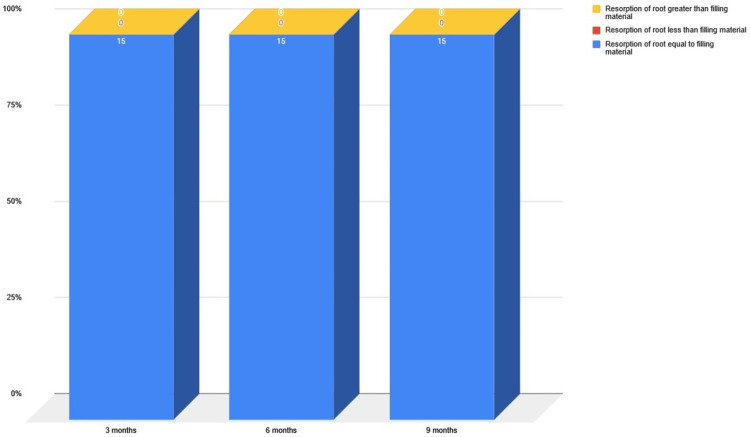
Graph showing the distribution of resorption of the filling material

Group B (Endoflas FS) showed 100% radiographic success with material resorbing at the same rate as the root in all cases at the end of three months, six months, and nine months (Figure 3).

**Figure 4:**
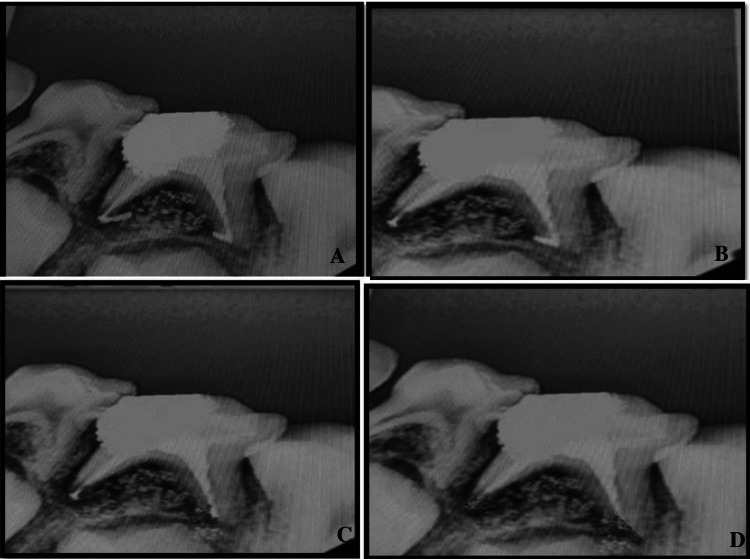
Radiographic evaluation of zinc oxide mixture (A) Immediate postoperative radiograph showing overfilling at root apex. (B) Postoperative radiograph at three months. (C) Postoperative radiograph at six months showing complete resorption of filling material. (D) Postoperative radiograph at nine months.

**Figure 5:**
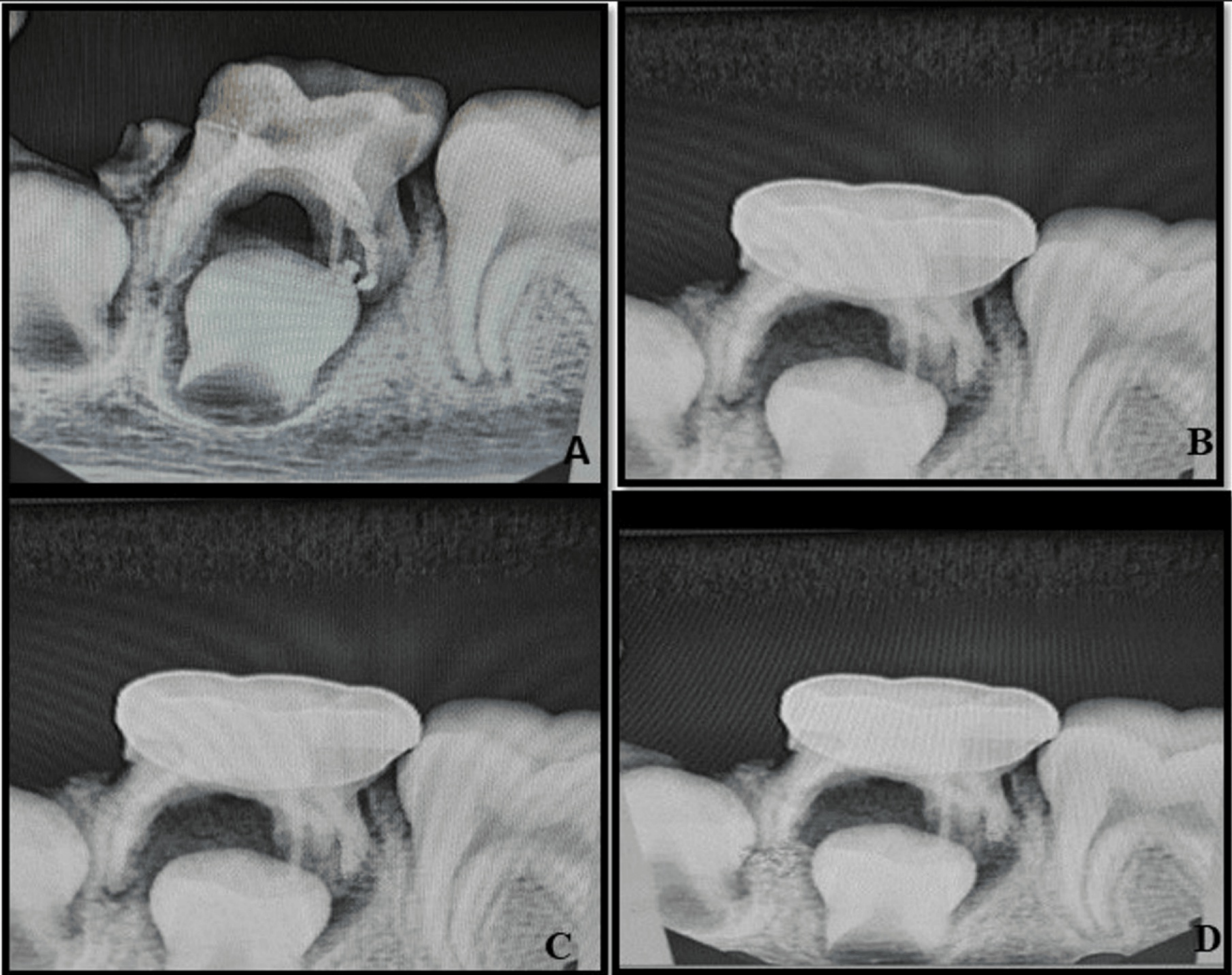
Radiographic assessment of Endoflas (A) Immediate postoperative radiograph showing overfilling at root apex. (B) Postoperative radiograph at three months. (C) Postoperative radiograph at six months. (D) Postoperative radiograph at nine months.

Table 2 represents the radiographic evaluation of overfilled material (Figures 4-5).

Group A showed that out of four over-obturated teeth, all showed complete resorption of extruded material at the end of three months. In group B, resorption of the excess material was seen in two teeth after three months, and the other two teeth showed resorption of the extruded material in six months. This shows that group B had a slower rate of resorption and lasted longer in the canals than group A. Based on the data analysis and calculation done by the chi-square test with SPSS version 24 software, a significant difference between the two groups was p < 0.001 (Table 2). The df was 1, and the effect size was 0.045.

**Table 2:** Distribution of resorption of over-pushed material in the groups in different follow-ups

Follow-up	Group A (n = 4)		Group B (n = 4)		p-value
	Resorbed	Not resorbed	Resorbed	Not resorbed	
Three months	4 (100.0%)	0 (0.0%)	2 (50.0%)	2 (50.0%)	<0.001
Six months	0 (0.0%)	0 (0.0%)	2 (100.0%)	0 (0.0%)	
Nine months	0 (0.0%)	0 (0.0%)	0 (0.0%)	0 (0.0%)	

Table 3 shows the overall comparison between the two groups. Both groups showed a 100% clinical success rate. Radiographically, group A showed an 80% success rate, whereas group B showed a 100% success rate at the end of nine months. Radiographically, group A showed a 100% success rate at the end of three months, 86.7% at the end of six months, and an 80% success rate at the end of nine months. Group B showed a 100% success rate at the end of nine months. The radiographic difference in success rate between the two groups was statistically significant (p < 0.05). The df was 1, and the effect size was 0.028.

**Table 3:** Comparison of overall success among both groups

Success parameters	Group A			Group B			p-value
	Three months n (%)	Six months n (%)	Nine months n (%)	Three months n (%)	Six months n (%)	Nine months n (%)	
								Radiological success	15 (100.0%)	13 (86.7%)	12 (80.0%)	15 (100.0%)	15 (100.0%)	15 (100.0%)	<0.05
Clinical success	15 (100.0%)	15 (100.0%)	15 (100.0%)	15 (100.0%)	15 (100.0%)	15 (100.0%)									

## Discussion

Primary teeth are important for phonetics, esthetics, and mastication [1]. It acts as a space maintainer during tooth eruption and establishes occlusion of permanent dentition [1,2,4]. A primary tooth also guides the eruption of the underlying permanent tooth [1,3,7]. Thus, it is important to preserve the position of primary teeth [2]. Zinc oxide eugenol is a traditional and commonly used endodontic filling material that shows different success rates in deciduous teeth (51%-92.3%) [10]. Over the past few years, the popularity of Endoflas as a root canal filling material has increased rapidly in pediatric dentistry [10,11]. 

In our study, we compared the clinical and radiographic observations of Endoflas with the mixture of zinc oxide, nHAp, and ozonated oil as an obturating material. Many studies have cited the success of Endoflas. Rewal et al. [6] observed a 100% success rate with Endoflas compared to zinc oxide eugenol, which showed an 84% success rate at the end of nine months, in favor of our study [6]. Ramar and Mungra et al. [12] concluded that Endoflas had a success rate of 95.1%, similar to the findings of our study, where we found a 100% success rate. It was reported that Endoflas showed healing ability and helped in bone regeneration [12].

Jeeva et al. [8] compared the cellular toxicity and antimicrobial effects of three materials and concluded that hap-fil, which consisted of 65% hydroxyapatite gel and iodoform as an antibacterial agent, showed better antimicrobial activity. Khadmi et al. [13], in their systematic review and meta-analysis, have reported that rotary instrumentation resulted in lesser postoperative pain in primary teeth. Chandra et al. [14] compared zinc oxide-ozonated oil and zinc oxide eugenol as an obturating material and concluded that our group A mixture was a better material than zinc oxide eugenol because of the beneficial properties of ozonated oil’s high viscosity, non-toxic nature, biocompatibility, antimicrobial nature, and wound healing property. Kottapalli et al. [15] conducted a study with a mixture of zinc oxide, nHAp, and saline as an obturating material, which showed a 66.6% radiographic success rate. 

Despite the success rate, its disadvantage is the eugenol content, which causes periapical irritation and toxicity [6,16-20]. Hence, we observed a radiographic and clinical success rate with the mixture of nHAp, zinc oxide, and ozonated oil. Nano hydroxyapatite mimics the enamel crystals, which helps in resorption along with physiologic resorption of teeth [17,21-23]. It is biocompatible, has a neutral pH, and is non-toxic [7,10,12,20]. All this encouraged us to use it as a vehicle [14,19,20,23]. Our result from the study regarding the success of Endoflas is in agreement with previous studies by Rewal et al. [6], Ramar and Mungra [12], which showed 100% clinical and radiographic success.

The limitation is that we did not segregate maxillary and mandibular teeth for the overall outcomes. The benefits of ozonated oil versus conventional saline in the mixture were not evaluated. The measurement of root canal length, proportional resorption rate, and ethnic variations among the study participants was not included. Hence, further clinical research with dedicated combinations on specific teeth, among different regions and ethnic children, would be needed to find an ideal obturating material for deciduous teeth.

## Conclusions

We have prospectively evaluated two different commercially available obturating materials and their clinical implications in primary dentition. From our study, we concluded that both Endoflas and the mixture of zinc oxide, nHAp, and ozonated oil showed good and equal clinical success rates. However, Endoflas showed better results and higher success rates radiographically at the end of three months, six months, and nine months compared to the mixture of zinc oxide, nHAp, and ozonated oil. Further clinical studies will shed light on this less explored topic in finding a potential obturating material for deciduous teeth.
